# Interactions and effects of a stannous-containing sodium fluoride dentifrice on oral pathogens and the oral microbiome

**DOI:** 10.3389/fmicb.2024.1327913

**Published:** 2024-02-15

**Authors:** Danyan Chen, Dillon Chew, Qianfeng Xiang, TzeHau Lam, Yajie Dai, Jiquan Liu, Lijiang Wang, Tao He, Ross Strand, Xiaolan Zhang, Linda Lim, Jian Xu, Yunming Shi, Weili Dong

**Affiliations:** ^1^State Key Laboratory of Oral and Maxillofacial Reconstruction and Regeneration, Key Laboratory of Oral Biomedicine Ministry of Education, Hubei Key Laboratory of Stomatology, School and Hospital of Stomatology, Wuhan University, Wuhan, China; ^2^Department of Periodontology, School and Hospital of Stomatology, Wuhan University, Wuhan, China; ^3^Department of Stomatology, Yiwu Central Hospital, Yiwu, Zhejiang, China; ^4^Singapore Innovation Center, The Procter & Gamble Company, Singapore, Singapore; ^5^Department of Dentistry-Regenerative Biomaterials, Radboud University Medical Center, Nijmegen, Netherlands; ^6^Single-Cell Center, CAS Key Laboratory of Biofuels and Shandong Key Laboratory of Energy Genetics, Shandong Energy Institute, Qingdao Institute of BioEnergy and Bioprocess Technology, Chinese Academy of Sciences, Qingdao, Shandong, China; ^7^Procter & Gamble Technology Co. Ltd, Beijing, China; ^8^The Procter & Gamble Company, Mason, OH, United States; ^9^Institute of Microbiology, Chinese Academy of Sciences, Beijing, China; ^10^College of Life Science, University of Chinese Academy of Sciences, Beijing, China

**Keywords:** oral microbiome, stannous-containing dentifrice, *in situ* biofilm, confocal laser scanning microscopy (CLSM), fluorescence *in situ* hybridization (FISH), 2bRAD-M

## Abstract

Numerous studies have investigated the effects of stannous ions on specific microbes and their efficacy in reducing dental plaque. Nonetheless, our understanding of their impact on the oral microbiome is still a subject of ongoing exploration. Therefore, this study sought to evaluate the effects of a stannous-containing sodium fluoride dentifrice in comparison to a zinc-containing sodium fluoride dentifrice and a control group on intact, healthy oral biofilms. Utilizing the novel 2bRAD-M approach for species-resolved metagenomics, and FISH/CLSM with probes targeting periodontal and caries associated species alongside Sn^2+^ and Zn^2+^ ions, we collected and analyzed *in situ* biofilms from 15 generally healthy individuals with measurable dental plaque and treated the biofilms with dentifrices to elucidate variations in microbial distribution. Although significant shifts in the microbiome upon treatment were not observed, the use of a stannous-containing sodium fluoride dentifrice primarily led to an increase in health-associated commensal species and decrease in pathogenic species. Notably, FISH/CLSM analysis highlighted a marked reduction in representative species associated with periodontitis and caries following treatment with the use of a stannous-containing sodium fluoride dentifrice, as opposed to a zinc-containing sodium fluoride dentifrice and the control group. Additionally, Sn^2+^ specific intracellular imaging reflected the colocalization of Sn^2+^ ions with *P. gingivalis* but not with other species. In contrast, Zn^2+^ ions exhibited non-specific binding, thus suggesting that Sn^2+^ could exhibit selective binding toward pathogenic species. Altogether, our results demonstrate that stannous ions could help to maintain a healthy oral microbiome by preferentially targeting certain pathogenic bacteria to reverse dysbiosis and underscores the importance of the continual usage of such products as a preventive measure for oral diseases and the maintenance of health.

## 1 Introduction

Dental plaques, which are naturally occurring complex biofilms in the oral cavity, are primarily composed of bacteria embedded in a three-dimensional extracellular matrix consisting of polysaccharides, proteins, lipids, and nucleic acids which provides for architecture and stability (Flemming and Wingender, 2010; Koo et al., 2013). Due to the warm, moist, and nutrient-rich conditions, the oral cavity serves as the optimum environment for biofilm formation with plaques tending to accumulate in hard-to-reach areas such as the gingival sulcus (Loe et al., 1930). The formation of oral biofilms is characterized by a systematic and dynamic process, commencing with the initial attachment of the early colonizers, predominantly gram-positive aerobes such as *Streptococcus gordonii* and *Streptococcus sanguinis*, followed by secondary colonization by filamentous anaerobic bacteria such as *Fusobacterium nucleatum* which acts as a “bridging species.” This is then followed by other anaerobes such as *P. gingivalis, Treponema denticola* and *Tannerella forsythia,* which tend to reside within the top layer of the biofilm (Socransky et al., 1998; Rosan and Lamont, 2000; Kolenbrander et al., 2006; Zijnge et al., 2010; Lasserre et al., 2018), thereby contributing to the development of a complex three-dimensional structure which defines the characteristic architecture of oral biofilms.

The oral microbiome is known to harbor over 700 bacterial species, with the majority maintaining a symbiotic relationship within the oral environment in a normal, healthy host (Aas et al., 2005; Kilian et al., 2016; Deo and Deshmukh, 2019; Radaic and Kapila, 2021). However, disruption of this balance due to external environmental factors (e.g., diet, stress, hormones, or poor oral hygiene habits) can shift the microbiome to a pathogenic state, resulting in microbial dysbiosis that leads to the development of oral diseases such as caries and periodontal disease (Marsh, 1994, 2003), which is prevalent globally (Nazir et al., 2020). For instance, excessive consumption of dietary sugars can lead to the fermentation of these carbohydrates into organic acids, thereby lowering the local pH which results in the selection for acidogenic species (e.g., *Streptococcus mutans*). Over time, the microbiome would shift toward a more cariogenic state, leading to the demineralization of the tooth enamel and subsequently irreversible dental caries (Pitts et al., 2017; Bhaumik et al., 2021). Likewise in periodontal diseases, accumulation of dental plaque as a result of poor oral hygiene represents another pathway to dysbiosis due to changes in the environment such as increased anaerobic conditions and gingival inflammation (Cobb and Killoy, 1990). Consequently, pro-inflammatory and anaerobic species gain a selective advantage and proliferate in this altered environment, leading to an escalation in inflammation which culminates in gingivitis, and eventually irreversible periodontitis if left untreated (Rosier et al., 2014). In both cases, substantial increases in the proportions of pathogenic species play a direct role in disease and thus health-maintaining mechanisms that prevent a shift to dysbiosis are essential in preventing and treating plaque-related dental diseases.

At present, good oral hygiene practices, such as regular toothbrushing with a dentifrice that contains active agents that control bacterial growth and accumulation (e.g., metal fluorides, quaternary ammonium compounds), are an effective way to maintain good oral health and prevent caries and periodontal diseases (Rajendiran et al., 2021). One such functional agent is Stannous Fluoride (SnF_2_), which has been extensively studied for its superior anti-caries effect and ability to reduce gingival inflammation and plaque in clinical settings (Makin, 2013; Biesbrock et al., 2019; Clark-Perry and Levin, 2020; Parkinson et al., 2020; Acherkouk et al., 2021; He et al., 2021). As an anti-caries agent, SnF_2_ provides protection by preventing demineralization of the tooth enamel and reducing plaque (Wefel et al., 2002; Stookey et al., 2004). While the exact mechanism of action for reducing gingivitis is not fully understood, it is believed that the stannous ion (Sn^2+^) exerts both a bactericidal and bacteriostatic effect by interfering with bacteria metabolism (Cvikl et al., 2018), inhibiting bacterial adhesion (Kirsch et al., 2019), and reducing plaque toxicity by binding to bacteria endotoxins (Haught C. et al., 2016; Haught J. C. et al., 2016). In addition, recent advancements in DNA and RNA sequencing technologies have provided new insights on how stannous could influence the tight interplay between the oral microbiota and oral health. For instance, metagenomic sequencing of supragingival plaques (He et al., 2021), *in situ* formed biofilms (Gumber et al., 2022), and saliva (Anderson et al., 2020; Kruse et al., 2021) has demonstrated a reduction in the proportion of disease-associated taxa and elevation of health-associated taxa upon the use of stannous-containing dentifrice, thus implying that stannous may also function to rebalance the oral microbiome by selectively targeting pathogenic species. These findings, however, have been limited to genus-level identification, which is of insufficient resolution for pinpointing or distinguishing the underlying microbial taxa that could be responsible for the observations.

While sequencing approaches could uncover the microbial complexity of oral biofilms, these come at the expense of destroying the biofilm’s spatial structure during nucleic acid extraction. Likewise, the use of *in vitro* biofilm models and *in vivo* plaque scraped from tooth samples may not accurately reflect the intra-oral physiological status and biofilm architecture, respectively (Zheng et al., 2015; Klug et al., 2016; Cheng et al., 2017). Consequently, despite numerous clinical studies investigating the effects of stannous ions on selected microbial species and their efficacy in reducing plaque in patients with oral diseases, the mechanisms by which these compounds influence the microbial community structure and spatial organization of intact oral biofilms in healthy individuals are still a subject of ongoing research. To address these challenges, fluorescence *in situ* hybridization (FISH) combined with confocal laser scanning microscopy (CLSM) on *in situ* biofilms provides a non-destructive method to visualize and quantify the three-dimensional spatial distribution of specific bacteria within oral biofilms (Al-Ahmad et al., 2007, 2009; Thurnheer et al., 2004). In this study, we seek to integrate a novel metagenomic sequencing method that enables species-level elucidation (Sun et al., 2022) with FISH/CLSM demonstrating species-level spatial distribution information to evaluate the effects of stannous-containing sodium fluoride dentifrice on the healthy oral microbiome and uncover new insights into its observed microbiome modulation properties.

## 2 Materials and methods

### 2.1 Collection and treatment of *in situ* biofilm

Fifteen adult subjects (*n* = 15), each of whom had given informed consent, between 18 to 60 years of age and in good general health were enrolled in a randomized, double-blind, single-center clinical trial in Wuhan, China. Subjects were required to have a minimum of 18 natural teeth, be in good oral health as confirmed through clinical examination by an experienced dentist, and with measurable dental plaque (score of 2–3 based on Turesky Modified Quigley Hein Plaque Index). Collection of the *in situ* plaque biofilm was achieved using 12 hydroxyapatite (HA) discs (Bei’erkang, Shanghai, China) fixed onto a customized removable oral splint (Supplementary Figure S1A) as described previously (Xiang et al., 2018). Subjects were instructed to wear the oral splint in the mouth except during meals (stored in an opaque container in humid conditions without soaking in liquid) and when performing oral hygiene procedures using a provided commercial dentifrice containing 0.243% NaF (Crest® Cavity Protection, Procter & Gamble, China) and a manual toothbrush (Crest® Velvet Soft Bristle, Procter & Gamble, China) twice daily over the course of the study. Subjects were also instructed to not use any other non-study oral hygiene products (e.g., mouth rinse, dental floss) during this time period. After the 48-h study duration, the biofilm coated HA discs were removed from the splint and processed without delay.

The 12 collected HA discs from each subject were then treated by transferring them (4 discs per treatment) into wells containing 500 μL of either (i) phosphate-buffered saline (PBS, negative control), (ii) 25% (v/v) paste suspension of a marketed stannous-containing sodium fluoride dentifrice (SN, Crest® Pro-Health toothpaste, Procter & Gamble, China), or (iii) 25% (v/v) paste suspension of a marketed zinc-containing sodium fluoride dentifrice (ZN) for 2 min without stirring. The suspensions were prepared prior to use by mixing 5 g of dentifrice with 15 mL of distilled water at 10,000 rpm for 10 min. Following which, the post-treatment biofilms were washed twice in PBS to remove residuals before being transferred into 1 mL PBS. 9 discs from each subject were stored at 4°C for microscopy analysis while the remaining 3 discs were frozen at −80°C for metagenomic analysis (Supplementary Figure S1B).

### 2.2 Genomic DNA extraction, library preparation, and metagenomic sequencing

The genomic DNA from each of the treated biofilms was first extracted using the TIANamp Micro DNA Kit (TIANGEN, Beijing, China). Following which, carrier RNA was added to enhance the yield, and the extracted genomic material was then eluted in 20 μL RNase-free water. Subsequently, the 2bRAD libraries were prepared following the protocol as described in previous studies (Lam et al., 2022; Sun et al., 2022). Briefly, 4 U of BcgI restriction enzyme (NEB, United States) was used to digest the genomic DNA at 37°C for 3 h. The resulting DNA fragments were then subjected to a ligation reaction with 0.2 μM of library-specific adaptors (Ada1, Ada2) at 4°C for 16 h, followed by heat inactivation of BcgI at 65°C for 20 min. Polymerase chain reaction (PCR) was performed on the ligated DNA using 7 μL ligated product, 0.1 μM primers, 0.3 mM dNTP, 1X Phusion HF buffer, and 0.4 U Phusion High Fidelity DNA polymerase (NEB, United States). The PCR conditions involved 16–28 cycles at 98°C for 5 s, 60°C for 20 s, 72°C for 10 s, and a final extension at 72°C for 10 min. The resulting libraries were purified using the QIAquick PCR purification kit (Qiagen, United States) and subsequently sequenced on the Illumina HiSeq X™ Ten platform. Library construction and sequencing were conducted by OE BioTech Co., Ltd., Qingdao, China.

### 2.3 Sequence processing and analysis

High-quality BcgI enzyme-digested sequence fragments of 32 bp were obtained after processing and cleaning the raw sequences. A total of 306.5 million reads were generated across the 45 samples with an average of 6.81 million reads per sample. Taxonomic profiling was performed using the 2bRAD-M computational pipeline available on Github: https://github.com/shihuang047/2bRAD-M. In summary, the reads were mapped against a pre-determined 2bRAD tag database which contains taxa-specific BcgI-derived sequences identified from 173,165 RefSeq microbial genomes. Reads coverage for each identified genome was calculated to estimate the relative abundance of each taxon. For reliable taxonomic profiling, taxonomic units that did not meet the following criteria were excluded from further analysis: (i) taxa-specific 2bRAD-tag number < 5, and (ii) sequenced reads number < 15. After applying these criteria, a total of 699 microbial species across the 45 samples remained in the dataset for subsequent analysis. All sequence data from this study have been submitted under Bioproject number PRJNA1031608 to NCBI and the sequencing profile of the samples are presented in Supplementary Table S1.

Library construction and sequencing of the control samples were conducted concurrently with the other samples. As a positive control, an internal mock community (Mock-CAS) consisting of five bacterial species (*Escherichia coli*, *Lactobacillus fermentum*, *Staphylococcus aureus*, *Streptococcus agalactiae*, and *Streptococcus mutans*) in equal proportions was used (Sun et al., 2022). Detailed taxonomic analysis highlighted the detection of all 5 species with *L. fermentum* being slightly overrepresented (Supplementary Table S2). In addition, two negative controls consisting of the DNA extraction mix were used to check against potential contamination. While species detected in the negative controls were primarily from the *Actinomyces*, *Neisseria*, *Ralstonia* and *Streptococcus* genus, the number of reads (NC1: 11.8 k, NC2: 22.5 k), were observed to be about 3 and 4 magnitudes lesser than the samples mean (6.8 million) and positive control, respectively (17.6 million), thus suggesting minimal background and/or cross-contamination (Supplementary Table S3).

Statistical analysis of the downstream taxonomy data was carried out using the R (v4.1.0) language (R Core Team, 2021). The R package “vegan” (v2.5.7) was used to calculate Alpha Diversity Indices and perform principal coordinates analysis (PCoA) ordination (with Bray–Curtis dissimilarity as the distance measure) based on the taxonomic abundance profiles of each sample in our dataset (Oksanen et al., 2020). Differences in community structure were evaluated by PERMANOVA test conducted with 999 permutations. As taxa abundances are not normally distributed, non-parametric tests were used with multiple testing correction where applicable. All other statistical tests and analyses were carried out with rstatix (Kassambara, 2021; v0.7.0) and all plots were built using ggplot2 (Wickham, 2016, p. 2; v3.3.5) and ggpubr (Kassambara, 2020; v0.4.0).

### 2.4 Fluorescence *in situ* hybridization

FISH was conducted following the protocol described by Amman and adapted for HA disks (Amann, 1995). In brief, the treated biofilms collected from the HA discs were first fixed in 4% paraformaldehyde in PBS (1.7 mM KH_2_PO_4_, 5 mM Na_2_HPO_4_ with 0.15 M NaCl, pH 7.2) at 4°C for 12 h. Following fixation, all samples were washed with PBS and incubated in a solution containing 50% (v/v) ethanol in PBS at 4°C for 12 h. The samples were then washed twice with PBS, and then incubated in the pre-hybridization solution containing 7 mg/mL lysozyme (105,000 U/mg; Fluka) in 0.1 M Tris/HCl (pH 7.2) and 5 mM EDTA at 37°C for 10 min to permeabilize cells in the plaque biofilm. The samples were then dehydrated with a series of ethanol washes (pre-chilled at −20°C) containing 50%, 80%, and 100% ethanol for 3 min each before being incubated with the probes for hybridization at a concentration of 50 ng each per 20 μL hybridization buffer (0.9 M NaCl, 20 mM Tris/HCl, 25% (v/v) formamide, and 0.01% (w/v) sodium dodecyl sulfate, pH 7.2) in 96-well plates (Costar) at 46°C for 2 h.

To study the different species involved in oral diseases, two combinations of four species-specific 16S rRNA-targeted oligonucleotide probes were used, with the first targeting periodontal pathogens vs. commensals, and the second for cariogenic bacteria vs. commensals. Spatial distribution of the bacteria species and metal ions in the biofilm were studied by further incubating the biofilm with Sn or Zn-specific probes alongside the 16S rRNA-targeted oligonucleotide probes. Upon hybridization, these biofilms were further incubated at 37°C for 30 min in a PBS solution containing 5 μM Sn or Zn-specific probes. All HPLC-purified species-specific oligonucleotide probes used in this study were synthesized commercially and labeled at the 5′-end with different fluorochromes (ThermoFisher, Shanghai, China). The combinations, sequences, 5′-modifications, and target species are listed in Table 1. After probe hybridization, the samples were subjected to incubation at 48°C for 15 min in a wash buffer (20 mM Tris/HCl, 5 mM EDTA, 159 mM NaCl and 0.01% (w/v) sodium dodecyl sulfate, pH 7.5) to eliminate non-specific binding and excess probes. Subsequently, the samples were rinsed with ice cold distilled water and allowed to air dry before being stored in 4°C for confocal microscopy.

**Table 1:** Probe combinations, sequences, 5′-modifications, and target species of used oligonucleotide probes in the different experiments.

Group	Probe	Sequence (5,–3,)	5, fluorochrome	Target	References
Periodontal	POGI	CAATACTCGTATCGCCCGTTATTC	Texas Red	*P. gingivalis*	Sunde et al. (2003)
	B(T)AFO	CGTATCTCATTTTATTCCCCTGTA	CY3	*T. forsythia*	Sunde et al. (2003)
	TRE II	GCTCCTTTCCTCATTTACCTTTAT	CY5	*T. denticola*	Moter et al. (1998)
	MIT588	ACAGCCTTTAACTTCAGACTTATCTAA	6-FAM	*S. oralis*	Thurnheer et al. (2004)
Cariogenic	MUT590	ACTCCAGACTTTCCTGAC	Texas Red	*S. mutans*	Paster et al. (1998)
	FUS664	CTTGTAGTTCCGCYTACCTC	CY5	*F. nucleatum*	Thurnheer et al. (2004)
	IF201	GCTACCGTCAACCCACCC	6-FAM	*A. naeslundii*	Foster and Kolenbrander (2004)
	MIT588	ACAGCCTTTAACTTCAGACTTATCTAA	ATTO 425	*S. oralis*	Thurnheer et al. (2004)
Sn distribution	Sn Probe R2	NA	Rhodamine B	Sn^2+^ ions	Lan et al. (2014)
	POGI	CAATACTCGTATCGCCCGTTATTC	CY5	*P. gingivalis*	Sunde et al. (2003)
	MIT588	ACAGCCTTTAACTTCAGACTTATCTAA	CY3	*S. oralis*	Thurnheer et al. (2004)
	S.s Probe	GCATACTATGGTTAAGCCACAGCC	ATTO 425	*S. sanguinis*	Li et al. (2014)
	EUB338	GCTGCCTCCCGTAGGAGT	6-FAM	*Eubacteria*	Al-Ahmad et al. (2007)
Zn distribution	Zinquin ethyl ester	NA	Zinquin	Zn^2+^ ions	NA
	POGI	CAATACTCGTATCGCCCGTTATTC	CY5	*P. gingivalis*	Sunde et al. (2003)
	MIT588	ACAGCCTTTAACTTCAGACTTATCTAA	CY3	*S. oralis*	Thurnheer et al. (2004)
	S.s Probe	GCATACTATGGTTAAGCCACAGCC	Texas Red	*S. sanguinis*	Li et al. (2014)
	EUB338	GCTGCCTCCCGTAGGAGT	6-FAM	*Eubacteria*	Al-Ahmad et al. (2007)

### 2.5 CLSM and image analysis

All samples were analyzed in 35 mm glass bottom dishes (Mattek) by confocal laser-scanning microscopy (TCS SP8 AOBS, Leica) using a 40X dry objective (HCX PL APO/bd.BL 40.0, Leica). Excitation of the FISH/metal fluorescent probes was performed at the following wavelengths: ATTO 425 and Zinquin ethyl ester, 405 nm; 6-FAM, 488 nm; CY3, 543 nm; R2, 561 nm; Texas Red, 594 nm; CY5, 633 nm, while fluorescence emission of the probes was measured at the following wavelengths: ATTO 425 and Zinquin ethyl ester, 450–500 nm; 6-FAM, 500–550 nm; CY3, 550-575 nm; R2, 575–600 nm; Texas Red, 600–650 nm; CY5, 650–700 nm. To minimize spectral overlap between the probes, CLSM was performed sequentially for each image. Each biofilm was scanned thrice at randomly selected positions away from the edges of the disks with Z-stacks generated by vertical optical sectioning from bottom to surface with step size of 1 μm. All confocal micrographs were processed to generate reconstructed 3D images using the Imaris (v10.0) software following steps similar to that in previous studies (Wilbert et al., 2020).

To quantify the biomass of the different target species within the *in situ* biofilms, the total fluorescent staining of the confocal micrographs was analyzed using the Leica LAS AF 3.3 image analysis program. The program was used to calculate the biofilm composition where a 100% of bacterial biomass was set as the total fluorescence intensity from the four channels of Z-stack images, while the relative biomass percentages of the targeted species were calculated using the fluorescence intensity of each channel relative to the total. This procedure was repeated three times across different locations to obtain the mean relative percentage for each species. Statistical analysis was performed using SPSS (IBM, 2013; v22.0) where the one-way ANOVA followed by Student-Neuman-Keuls *post hoc* test were used to calculate significant differences in mean relative percentage of individual species upon dentifrice treatment.

## 3 Results

### 3.1 Effects of dentifrice treatment on oral microbiome balance

**Figure 1:**
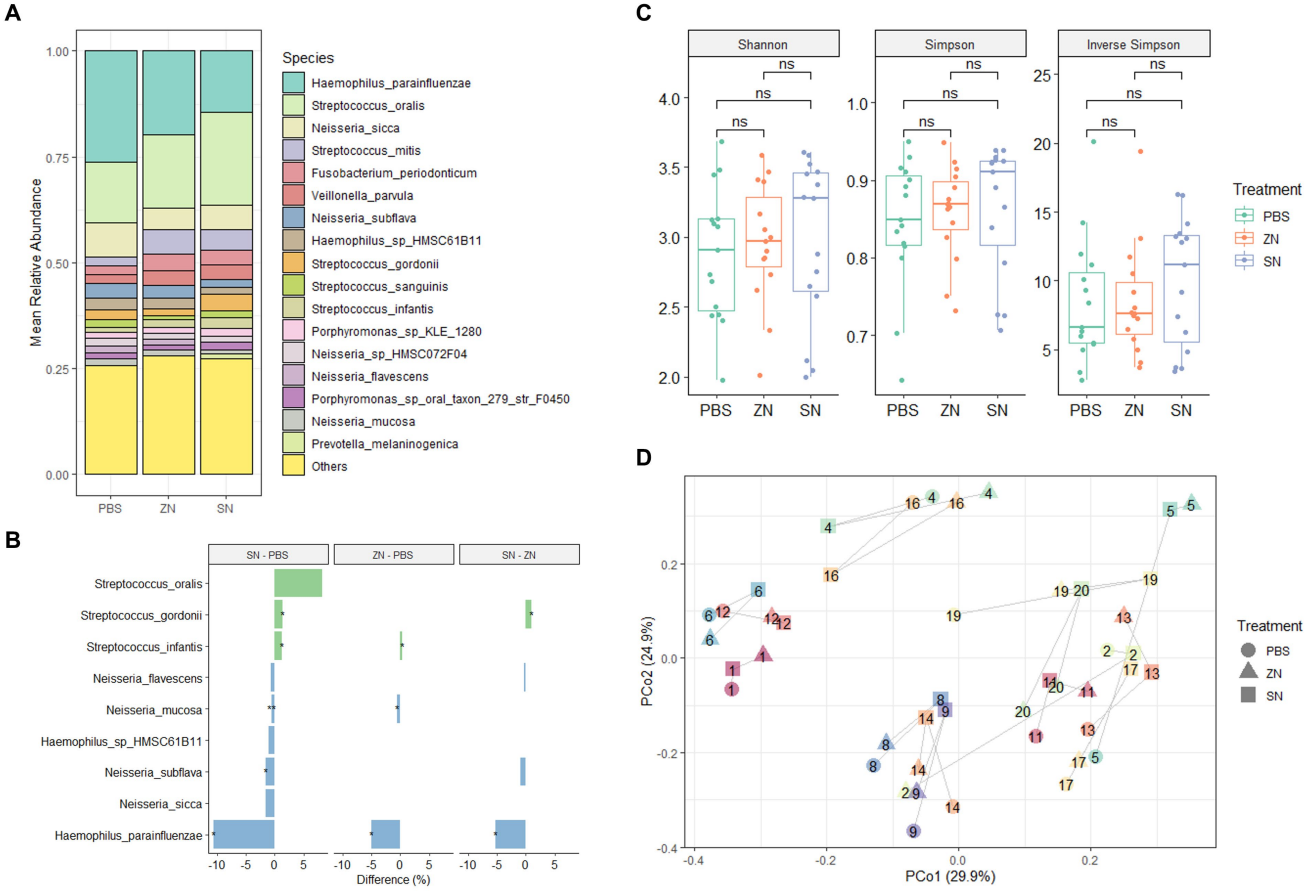
Species-level microbiome analysis of the *in situ* biofilms: **(A)** Mean relative abundance of the identified species across all samples (*n* = 15) for the three treatment groups combined. Species with mean relative abundance of <0.01 (1%) were grouped under “Others.” **(B)** Differential abundance of top species (mean relative abundance > 0.01 (1%), with *p* < 0.1, FDR-adjusted paired-sample Wilcoxon test, FDR-adjusted) across the treatment groups. Species which were higher after treatment are shown in green while those that reduced are shown in blue. ^*^p ≤ 0.05; ^**^p ≤ 0.01. **(C)** No pairwise significant difference (*p* < 0.05) was observed between the three treatment groups for the Shannon, Simpson, and Inverse Simpson index. Each point on the boxplot corresponds to a sample. The *p-*values shown above the boxplots were derived using the paired-sample Wilcoxon test: *ns*, not significant. **(D)** Principal coordinates analysis (PCoA) ordination plot based on Bray-Curtis distance revealed that the microbial composition did not differ based on treatment group (PERMANOVA; *R*^2^ = 0.042, *p* = 0.508), but rather by subject (PERMANOVA; *R*^2^ = 0.769, *p* = 0.001). Each point corresponds to a sample, is colored, number labeled and connected by gray lines according to the subject ID and has a shape according to the treatment group.

To investigate the impact of stannous on the oral microbiome, the 2bRAD-M sequencing approach was used to compare the biofilm composition treated with a stannous-containing sodium fluoride dentifrice (SN) against a zinc-containing sodium fluoride dentifrice (ZN). At the phylum level, the dominant taxa across the three treatment groups were *Proteobacteria* (47.1%), *Firmicutes* (40.3%), *Bacteroidetes* (5.7%), *Fusobacteria* (3.6%) and *Actinobacteria* (3.2%), which represented over 99% of the microbiome in each sample (Supplementary Figure S2A). Consistent with previous investigations of *in situ* oral microbiomes (Wake et al., 2016; Conrads et al., 2019; Gumber et al., 2022), analysis at the genus level revealed that *Streptococcus* (33.0%), *Haemophilus* (25.2%), *Neisseria* (20.5%), *Veillonella* (5.6%), *Fusobacterium* (3.4%), *Porphyromonas* (3.0%), *Rothia* (2.5%), and *Prevotella* (1.8%) were the predominant taxa shared among all samples. Collectively, these accounted for approximately 93% of the overall microbiome composition (Supplementary Figure S2B). Furthermore, comparison of SN and ZN treatment against the control at the genus level highlighted significant differences in only *Haemophilus* (SN: −14.7%, *p* = 0.010; ZN: −7.2%, *p* = 0.035), *Neisseria* (SN: −7.8%, *p* = 0.010; ZN: −4.9%, *p* = 0.055 – marginal) and *Streptococcus* (SN: 15.1%, *p* = 0.003; ZN: 6.7%, *p* = 0.053 – marginal). Of the 699 identified taxa at the species level, the five most abundant species (Figure 1A) were *Haemophilus parainfluenzae* (20.1%), *S. oralis* (17.9%), *Neisseria sicca* (6.2%), *Streptococcus mitis* (4.3%), and *Fusobacterium periodonticum* (3.1%). These species, which are typically considered as oral commensals (Querido and De Araujo, 1976; Slots et al., 1983; Baty et al., 2022; Perera et al., 2022), constituted more than 50% of the microbiome. In all, only 31 species from the *Haemophilus*, *Neisseria*, *Prevotella*, *Streptococcus*, *Schaalia* and *Veillonella* genus were observed to have significantly different abundance between the treatment groups (Supplementary Table S4). Notably, the opportunistic pathogen *H. parainfluenzae* was significantly reduced where a difference of 5.1% (*p* = 0.035) and 10.6% (*p* = 0.013) as compared to the control group was observed for the ZN and SN treatment groups, respectively (Figure 1B). SN treatment also led to a pronounced increase in the commensal *S. oralis* by 8.2%, albeit marginally significant (*p* = 0.054), as well as in *S. gordonii* at 1.3% (*p* = 0.038) when compared to the control group. However, there was no observed difference in response to ZN treatment.

From a community standpoint, there was no significant difference in alpha-diversity (Shannon, Simpson, and Inverse Simpson indexes) across the three treatment groups (*p* > 0.05, Figure 1C). In addition, low beta-diversity values (average Bray-Curtis Distance of 0.3, Supplementary Figure S3) between the control and treatment groups were also observed, suggesting that the microbial communities in all three groups were fairly similar. This was further supported by PCoA analysis (Figure 1D), which showed that the samples were fairly well-distributed in ordination space and did not cluster by treatment group (PERMANOVA; *R*^2^ = 0.042, *p* = 0.508). However, clusters were mainly observed among samples collected from the same individual (PERMANOVA; *R*^2^ = 0.769, *p* = 0.001). Collectively, these observations suggest that while SN dentifrice treatment did not cause significant shifts in the bacterial community structure, both the increase in commensals and reduction in pathogenic species could still be observed, thus preserving the natural healthy oral microbiome.

### 3.2 Changes in the ratio of pathogenic bacteria *in situ* biofilm following dentifrice treatment

**Figure 2:**
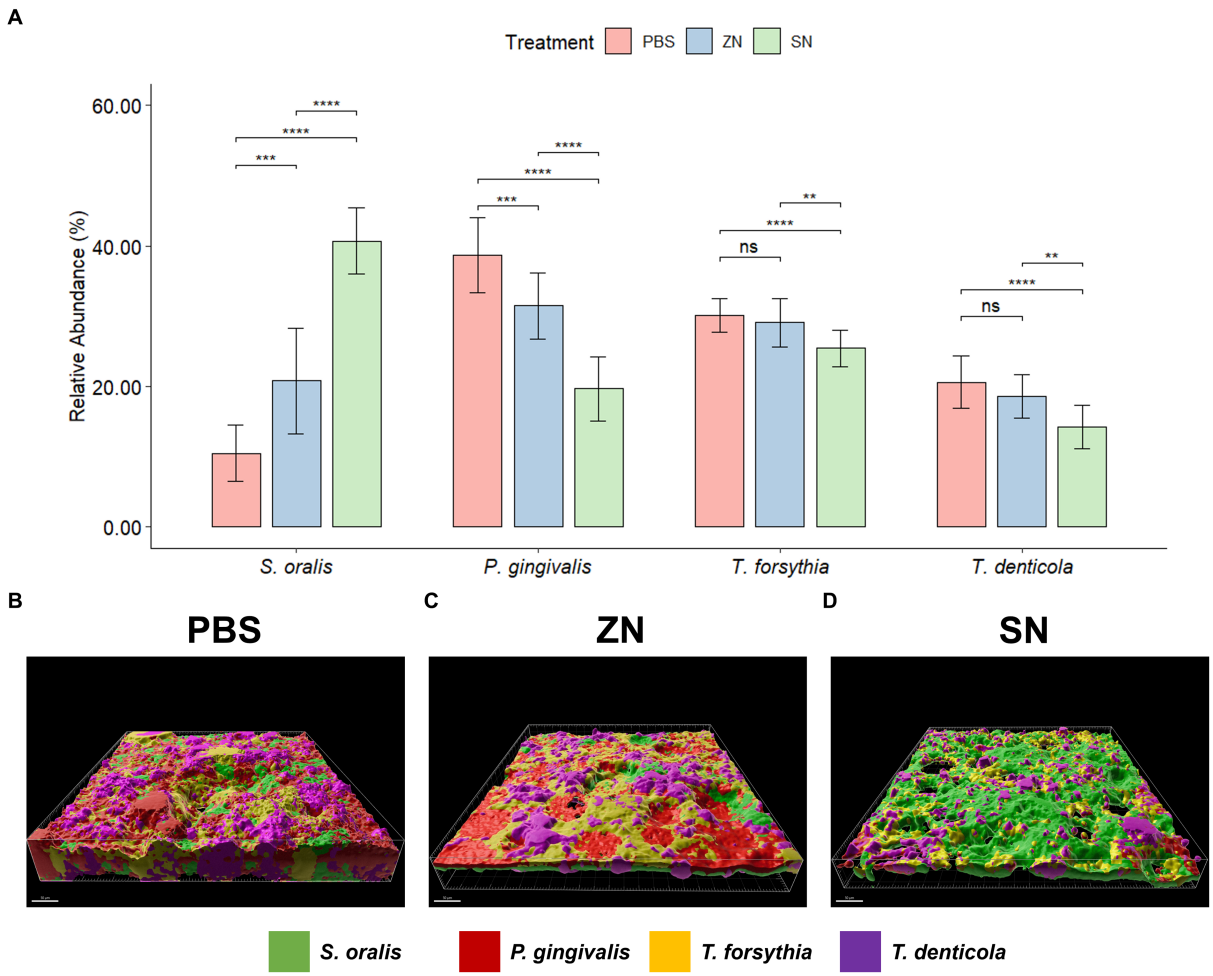
Representative 3D reconstructed FISH-CLSM images and biofilm composition analysis for periodontal related species. **(A)** Composition of the biofilm where the mean abundance (*n* = 15) of each targeted species is expressed as a percentage of the total. Error bars in the bar graphs represents SD and comparisons between treatments are shown with brackets above the bars with statistical significance as follow: *ns*: not significant, ^*^p ≤ 0.05, ^**^p ≤ 0.01, ^***^p ≤ 0.001, ^****^p ≤ 0.0001. Simultaneous hybridization with species-specific probes targeted toward commensals and periodontal related species: MIT588 (green) for *S. oralis*, POGI (red) for *P. gingivalis*, B(T)AFO (yellow) for *T. forsythia*, and TRE II (purple) for *T. denticola* on the *in-situ* biofilms treated with **(B)** PBS, **(C)** ZN, and **(D)** SN. Bar: 50 μm.

**Figure 3:**
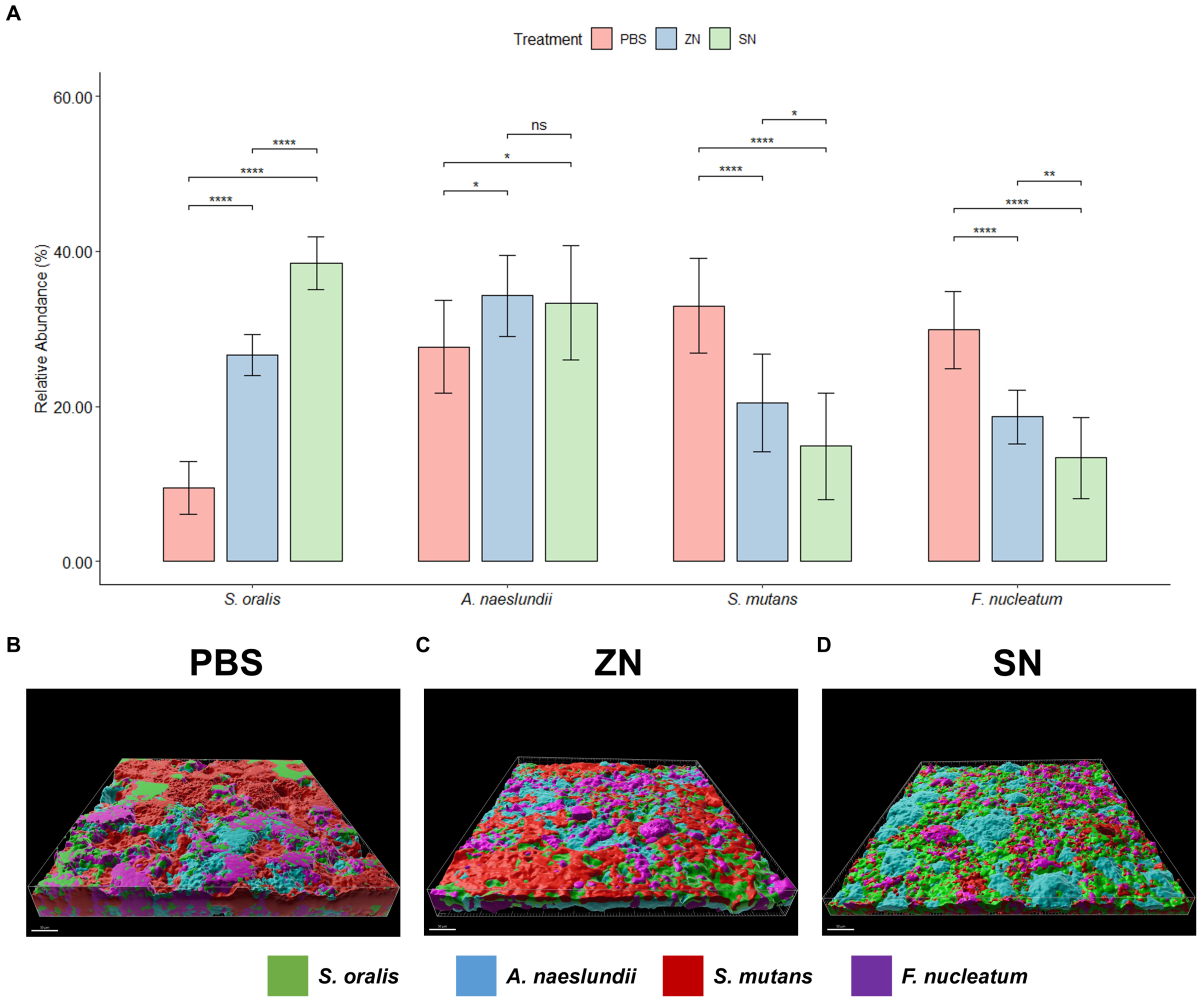
Representative 3D reconstructed FISH-CLSM images and biofilm composition analysis for caries related species. **(A)** Composition of the biofilm where the mean abundance (*n* = 15) of each targeted species is expressed as a percentage of the total. Error bars in the bar graphs represents SD and comparisons between treatments are shown with brackets above the bars with statistical significance as follow: *ns*: not significant, ^*^p ≤ 0.05, ^**^p ≤ 0.01, ^***^p ≤ 0.001, ^****^p ≤ 0.0001. Simultaneous hybridization with species-specific probes targeted toward commensals and caries related species: MIT588 (green) for *S. oralis,* IF201 (blue) for *A. naeslundii*, MUT590 (red) for *S. mutans*, and FUS664 (purple) for *F. nucleatum* on the *in-situ* biofilms treated with **(B)** PBS, **(C)** ZN, and **(D)** SN. Bar: 50 μm.

While 2bRAD-M was able to provide an overview of the microbial community, species-specific FISH was used for targeted visualization of key bacterial species related to periodontal disease and caries, and the representative 3D reconstructed CLSM z-stack images along with quantitative results are presented in Figures 2, 3. Selecting for periodontal species (Figure 2A), the mean relative percentage (± SD) of *P. gingivalis* was observed to decrease significantly (*p* < 0.05) in comparison to the PBS control group in both the ZN and SN treatment groups (31.48% ± 4.67 and 19.65% ± 4.63% respectively, vs. 38.71% ± 5.33%). On the other hand, a significant difference (*p* < 0.05) was only observed between the control group and SN treatment for both *T. forsythia* (25.46% ± 2.61% vs. 30.16% ± 2.37%) and *T. denticola* (14.22% ± 3.13% vs. 20.60% ± 3.72%). Among the cariogenic species (Figure 3A), a significant decrease (*p* < 0.05) relative to the PBS control group in both ZN and SN treatments was observed for both *S. mutans* (20.45% ± 6.33 and 14.84% ± 6.90% respectively, vs. 32.95% ± 6.12%) and *F. nucleatum* (18.65% ± 3.46 and 13.32% ± 5.28% respectively, vs. 29.88% ± 4.99%). Both ZN and SN treatments showed a significant increase (*p* < 0.05) in *A. naeslundii* relative to the PBS control group (34.26% ± 5.27 and 33.34% ± 7.34%, vs. 27.65% ± 5.99%). As for the commensal *S. oralis* which were present in both treatment groups, its mean relative percentage in both ZN and SN treatments was observed to have increased (*p* < 0.05) relative to the PBS control group in both the periodontal targeted (ZN: 20.80% ± 7.51%, SN: 40.67% ± 4.70%, vs. PBS: 10.50% ± 4.06%) and cariogenic targeted (ZN: 26.64% ± 2.64%, SN: 38.50% ± 3.39%, vs. PBS: 9.51% ± 3.41%) experiments. Statistical differences between the ZN and SN treatments were also observed for all species except for *A. naeslundii*. For all periodontal (*P. gingivalis*, *T. forsythia*, *T. denticola*) and cariogenic (*S. mutans*, *F. nucleatum*) species, the mean relative percentage after SN treatment was significantly lower (*p* < 0.05) than that of the ZN treatment. On the contrary, SN treatment showed a significant increase (*p* < 0.05) in the oral commensal *S. oralis* in both experiments as compared to the ZN treatment. Altogether, these data suggest that SN treatment was more effective than ZN treatment in reducing the abundance of periodontal and cariogenic species, in addition to increasing the abundance of oral commensals.

### 3.3 Stannous ions in stannous-containing sodium fluoride dentifrice selectively binds to *P. gingivalis*

**Figure 4:**
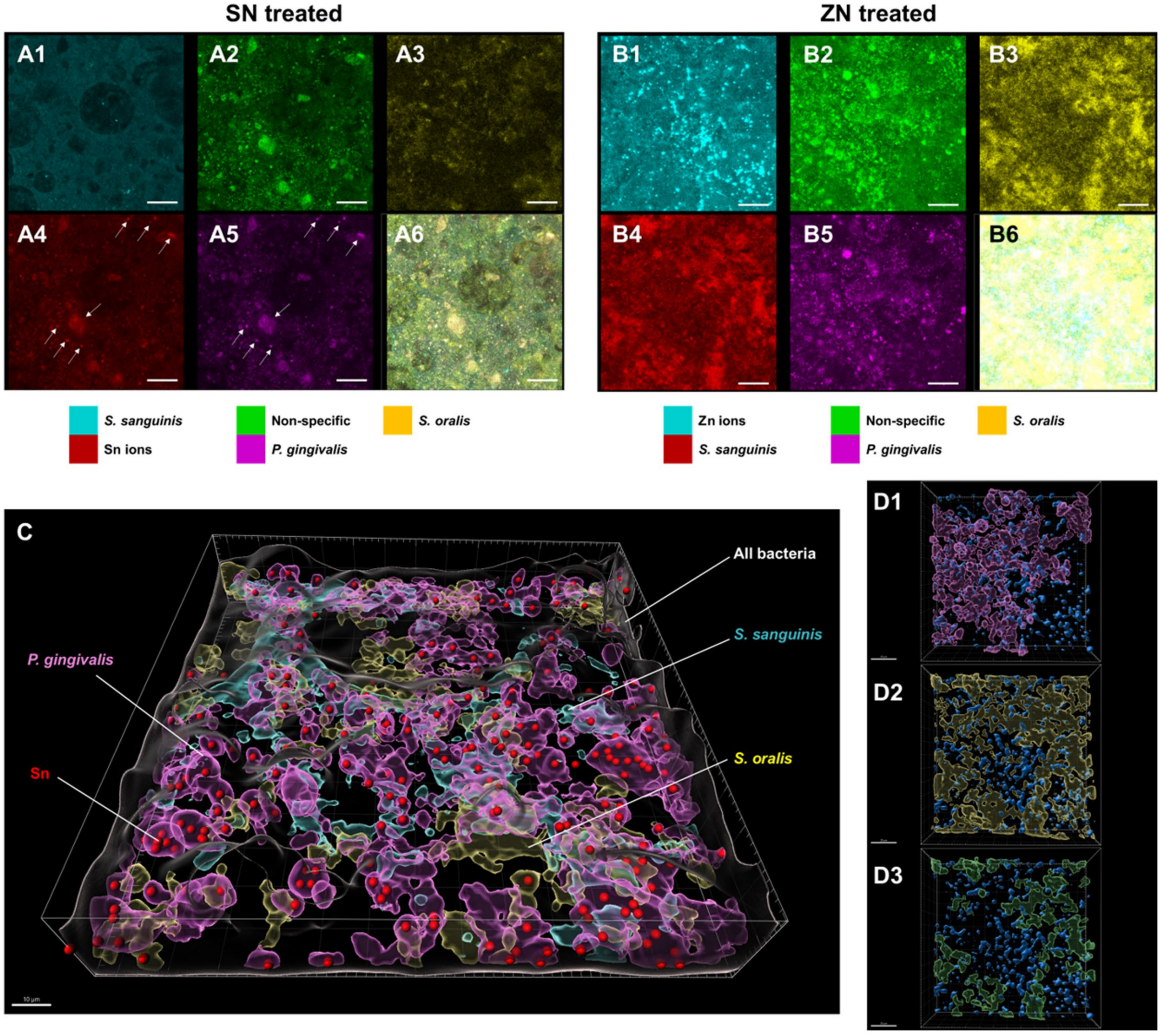
Representative FISH-CLSM and 3D reconstructed images using probes targeting specific bacterial strains and metal ions. Simultaneous hybridization for the SN treated biofilm with species-specific probes targeted for: **(A1)** *S. sanguinis* (cyan), **(A2)** non-specific bacteria (green), **(A3)** *S. oralis* (yellow), **(A4)** Sn^2+^ (red) and **(A5)** *P. gingivalis* (purple). **(A6)** Superimposed image from all channels. Arrows in **(A4,A5)** indicate the colocalization of Sn^2+^ with *P. gingivalis*. Bar: 20 μm. Simultaneous hybridization for the ZN treated biofilm with species-specific probes targeted for: **(B1)** Zn^2+^ (cyan), **(B2)** non-specific bacteria (green), **(B3)** *S. oralis* (yellow), **(B4)** *S. sanguinis* (red), and **(B5)** *P. gingivalis* (purple). **(B6)** Superimposed image from all channels. **(C)** Combined 3D reconstructed image for SN-treated *in situ* biofilm with probes targeting for Sn^2+^ (red), *S. oralis* (yellow), *S. sanguinis* (cyan), *P. gingivalis* (purple) and non-specific white (non-specific). Bar: 10 μm. **(D)** 3D reconstructed images highlighting the spatial distribution of Zn^2+^ (blue) along with bacterial species of **(D1)** *P. gingivalis*, (purple), **(D2)** *S. oralis* (yellow), and **(D3)** *S. sanguinis* (green) in the treated biofilms. Bar: 20 μm.

To elucidate the observed differences between SN and ZN treatments, additional fluorescent probes that specifically bind to Sn^2+^ and Zn^2+^ ions were used to further investigate the interactions between these ions and the bacteria species in the *in-situ* biofilm. For the SN treated biofilm, colocalization of the Sn^2+^ specific probes was only observed with the probes specific to the pathogenic species of *P. gingivalis* (Figure 4A) while no colocalization of Sn^2+^ probes with the commensal species of *S. oralis* and *S. sanguinis* was observed. In contrast, no colocalization of the Zn^2+^ probes with any of the targeted species was observed for the ZN treatment (Figure 4B). Spatial analysis of the biofilm was performed using 3D reconstructed CLSM z-stack images. Within the biofilm, late colonizers such as *P. gingivalis* were observed to be located in upper layer of biofilm while early colonizers such as *S. oralis* and *S. sanguinis* were mainly located in bottom layer of biofilm (Figure 4C). Notably, SN treatment revealed a pronounced accumulation of Sn^2+^ ions primarily within the pathogenic species *P. gingivalis*, especially within larger bacterial clumps, while showing no accumulation in the commensal species *S. oralis* and *S. sanguinis*. In contrast, there was no clear colocalization of Zn^2+^ ions upon ZN treatment with any of the bacterial species in the 3D images (Figure 4D). In all, these observations suggest that Sn^2+^ exhibits a selective binding toward *P. gingivalis*, a key pathogenic species implicated in periodontitis development, and that this selective pathogen binding mechanism could be unique to Sn^2+^ as compared to Zn^2+^.

## 4 Discussion

Stannous ions in oral hygiene products such as dentifrices have a long history of use for oral health improvement by preventing the dysbiosis of the oral microbiome, which could otherwise lead to the development of caries and periodontal diseases. While a considerable body of literature has proven the clinical benefits of stannous, its mode of action in restoring balance to the oral microbiome is not yet fully understood. Moreover, the ways in which these compounds impact the overall microbial community structure and spatial arrangement within undisturbed oral biofilms of healthy individuals remain unclear. Thus, the purpose of the present investigation was to further examine the mechanisms of stannous ions on intact, healthy oral biofilms. To achieve this, we employed a combination of *in situ* plaque models within the oral cavity and *ex vivo* dentifrice supernatant immersion methods to preserve the biofilm’s three dimensional structure. Following which, the two complementary approaches of metagenomics and FISH/CLSM analysis were used to shed light on the interactions between stannous ions and the oral microbiome.

In contrast to prior metagenomic studies of the oral microbiome via 16S methods or shotgun methods (Xiao et al., 2016; Chen et al., 2018; Conrads et al., 2019; Gumber et al., 2022), this study is the first to use 2bRAD-M, a metagenome reduction sequencing approach which enables species-resolved profiling of oral microbiomes. Notably, the obtained microbiome profiles were consistent with previous findings at the genus level, while additionally offering the advantage of enhanced resolution for the identification of microbial species relevant to oral diseases. Although healthy individuals were sampled, interestingly, species implicated in periodontal diseases, such as *F. nucleatum* (mean abundance = 0.23%, prevalence = 75.6%) and *P. gingivalis* (mean abundance = 0.002%, prevalence = 64.4%), were present at low abundance but in high prevalence, thus suggesting that the ratio of pathogenic bacteria in the oral biofilms, rather than detection, plays a pivotal role in plaque pathogenicity in healthy individuals. While there were some differences in the relative abundances of certain commensals and pathogenic species upon SN and ZN treatments, no significant changes in the overall diversity of the microbiome were observed in single 2-min treatment that is representative of a typical consumer toothbrushing behavior. It can be argued that maintaining similar levels of diversity following treatment is crucial for individuals’ oral health, as deviations from a healthy oral microbiome often result in a dysbiotic state that may eventually lead to disease. Consequently, major shifts in the oral microbiome upon treatment, are expected in diseased individuals, those with sub-optimal health (He et al., 2021), and/or after multiple treatments. This study also reveals high microbial similarity between samples collected from the same individual despite treatment, and thus it is probable that other individual factors such as oral hygiene practices and diet play a larger role in shaping the oral microbiome. Collectively, these results corroborate and deepen the findings from previous metagenomic studies on the oral microbiome (Anderson et al., 2020; Kruse et al., 2021; Gumber et al., 2022), which indicate that stannous containing oral hygiene products do not induce a substantial alteration in the oral microbiome of healthy individuals, thereby preserving the natural and healthy microbiota.

Having observed minimal alterations to the microbiome, our subsequent investigation focused on examining the ratio and spatial distribution of specific bacterial strains pertinent to oral diseases within the oral biofilm with a targeted method. Given the low relative abundances of oral pathogens observed in the metagenomics dataset, FISH/CLSM was used to investigate further as it is a much more sensitive technique. To achieve this, two distinct sets of FISH probe combinations which reflect the microbial dynamics associated with periodontal disease and dental caries were designed. In the context of periodontal disease, the probes targeted three members of the pathogenic “red complex,” *P. gingivalis*, *T. forsythia*, and *T. denticola* (Socransky et al., 1998), alongside the beneficial commensal *S. oralis*, thereby representing the interplay between the pathogens responsible for periodontal disease and the beneficial commensals within the microbiota. Similarly, in the context of dental caries, the probes targeted the primary causative agent *S. mutans*, the pivotal “bridging species” *F. nucleatum*, and two beneficial commensals, *S. oralis* and *A. naeslundii*. By using three-dimensional reconstructions of confocal laser scanning microscopy (CLSM) images, we were able to preserve the biofilm structure and visualize the spatial distribution of different targeted species in the *in-situ* biofilm. Our findings were consistent with the spatiotemporal model of oral bacterial colonization proposed by Kolenbrander et al., which suggests that early colonizers, such as *S. oralis*, inhabited the lower layers close to the tooth surface, while members of the red complex such as *P. gingivalis* are primarily found at the upper layer of the biofilm (Kolenbrander et al., 2006). Additionally, this study also reaffirms the penetration of stannous ions into the biofilm, as previously demonstrated (Xiang et al., 2018), and unveiled the additional insight of stannous accumulation primarily within *P. gingivalis* to drive its anti-gingivitis properties. With an elevation in *S. oralis* levels post SN treatment observed in both metagenomics and FISH/CLSM analysis, taken together, these results suggest that stannous ions exhibit selective binding and targeted antibacterial activity toward pathogenic bacteria over beneficial commensal species. Unlike zinc, which is another conventional broad spectrum active with antibacterial efficacy, these findings suggests that the selectivity of stannous results in a higher proportion of beneficial bacteria within the microbiota, thus aiding in maintaining periodontal health.

Based on our findings, we suggest a possible mode of action that could elucidate how stannous facilitates the rebalancing of the oral microbiome. Upon dentifrice application, a chemical gradient is created across the biofilm in which the upper layer has a higher exposure to stannous ions upon contact, and thus a greater uptake by the pathogenic species (“red complex”) such as *P. gingivalis* which typically reside there. Since stannous ions exhibit a pronounced binding affinity for lipopolysaccharides (LPS), which constitute a critical component of the cell wall in gram-negative bacteria, it is highly probable that stannous ions would become bound to LPS and thus have a more pronounced effect on the gram-negative pathogenic species (Haught J. C. et al., 2016). This LPS-Sn binding phenomenon distinguishes stannous ions from other antibacterial agents like triclosan and zinc, which do not demonstrate LPS binding. Given the sustained antimicrobial properties of stannous including its metabolism inhibition (Cvikl et al., 2018; Shi et al., 2018) and EPS (extracellular polysaccharides) reduction effects (Cheng et al., 2017; Gumber et al., 2022), it is likely that the structure and microbial community at the top layer of the biofilm would be impacted to a greater extent. However, it is important to note that stannous bioavailability and antimicrobial efficacy is influenced by formulation (He et al., 2020) and source (Tobler et al., 2023), thus implying that the outcomes observed for one particular formula may not be universally applicable to all stannous-containing dentifrice. While stannous is able to penetrate into the biofilm (Xiang et al., 2018), the susceptibility of antimicrobials toward oral microbes can vary. For instance, *Neisseria* species have been reported to be suppressed by a wide range of antimicrobial agents, while viridans streptococcal species such as *S. mitis* and *S. oralis* are marginally susceptible (Donnelly et al., 2015). In addition, past studies have shown that stannous ions do not have the same antibacterial inhibitory effect across strains. Notably, a stannous fluoride-containing dentifrice have been reported to exhibit lower MIC (minimum inhibitory concentration) levels *in vitro* against periodontal and cariogenic pathogens such as *P. gingivalis*, *F. nucleatum*, and *S. mutans,* compared to other microbes such as *B. subtilis* and *S. aureus* (Haraszthy et al., 2010). Therefore, it is plausible that while stannous has a broad-spectrum antibacterial activity, it could additionally exhibit selectivity against certain pathogenic species which would facilitate the reversing of dysbiosis. Nonetheless, the underlying molecular mechanisms regarding the selectivity of stannous, and its efficacy against other pathogenic species implicated in oral diseases (e.g., *T. denticola*, *T. forsythia*, *S. mutans*) would need to be further investigated and could be done through additional transcriptomic or proteomic approaches.

It is also important to note that the detection of species implicated in oral diseases from both metagenomic sequencing and DNA hybridization methods in our study does not suggest disease susceptibility as these data do not provide definitive information about their viability or virulence, and samples were obtained from healthy individuals. Furthermore, the values reported in this study are relative abundances, thus it is not definite if an observed difference in relative abundance between treatments is due to an increase in certain species, or an overall decrease in others. While these results also suggest that host factors and behavior, rather than treatment, are major factors that shape the oral microbiome, the long-term effect of such products and its influence on the host gingival cells needs to be further investigated. Future larger scale studies utilizing spatial-omics and microbial single-cell technologies while taking into account of biofilms from patients with caries and/or periodontitis can better illustrate the complex microbial dynamics at the micro and macroscale with higher resolution while maintaining the structural integrity of the biofilm (Borisy and Valm, 2021; Dar et al., 2021; Xu et al., 2023) to provide a more comprehensive understanding of the impact of different treatments on oral biofilms.

## 5 Conclusion

In summary, this study was the first to adopt the 2bRAD-M technology coupled with direct visualization of intact oral biofilms with the FISH/CLSM technique to provide further insights on how treatment with a stannous-containing sodium fluoride dentifrice could reverse dysbiosis and better maintain the healthy oral microbiome. Unlike conventional broad spectrum antibacterial actives such as zinc, our results show that stannous ions could help to maintain the healthy oral microbiome by preferentially targeting pathogenic bacteria such as *P. gingivalis*. The elevation of oral commensals along with the relative reduction of species associated with periodontitis and caries without causing major shifts in the microbiome under the experimental conditions underscores the importance of the habitual usage of such products as a preventive measure for oral diseases and maintenance of health.

## Data availability statement

All sequencing data generated in this study were deposited at NCBI under BioProject number PRJNA1031608.

## Ethics statement

The studies involving humans were approved by Ethics Committee of School and Hospital of Stomatology, Wuhan University (IRB: CSD2022019). The studies were conducted in accordance with the local legislation and institutional requirements. The participants provided their written informed consent to participate in this study.

## Author contributions

DaC: Conceptualization, Data curation, Formal analysis, Investigation, Methodology, Software, Writing – review & editing. DiC: Data curation, Formal analysis, Investigation, Methodology, Software, Writing – original draft. QX: Data curation, Investigation, Methodology, Software, Writing – review & editing. TL: Data curation, Formal analysis, Investigation, Methodology, Software, Supervision, Writing – review & editing. YD: Data curation, Investigation, Methodology, Writing – review & editing. JL: Funding acquisition, Project administration, Resources, Supervision, Writing – review & editing. LW: Funding acquisition, Project administration, Resources, Supervision, Writing – review & editing. TH: Funding acquisition, Project administration, Resources, Supervision, Writing – review & editing. RS: Funding acquisition, Project administration, Resources, Supervision, Writing – review & editing. XZ: Data curation, Investigation, Writing – review & editing. LL: Funding acquisition, Project administration, Resources, Supervision, Writing – review & editing. JX: Funding acquisition, Project administration, Resources, Supervision, Writing – review & editing. YS: Conceptualization, Methodology, Supervision, Writing – review & editing, Project administration, Software. WD: Conceptualization, Funding acquisition, Methodology, Supervision, Writing – review & editing, Project administration, Resources.
