# Supplementary material for: Interactions and effects of a stannous-containing sodium fluoride dentifrice on oral pathogens and the oral microbiome
Source: Front Microbiol. 2024 Feb 15;15:1327913. doi: 10.3389/fmicb.2024.1327913 (PMC10902866; doi:10.3389/fmicb.2024.1327913)
Supplement: Supplementary file 1 [file Data_Sheet_1.DOCX]

*Supplementary Materials for* Interactions and effects of Stannous-containing Sodium Fluoride dentifrice on oral pathogens and the oral microbiome

Danyan Chen^1,2,3^, Dillon Chew^4^, Qianfeng Xiang^5^, TzeHau Lam^4^, Yajie Dai^6^, Jiquan Liu^4^, Lijiang Wang^7^, Tao He^8^, Ross Strand^4^, Xiaolan Zhang^9^, Linda Lim^4^, Jian Xu^6,10^, Yunming Shi^7^, Weili Dong^1,2*^

# Supplementary Tables and Figures

## Supplementary Tables

Please see attached excel sheet for Tables S1-4

Table S1: Sequencing profile of samples

Table S2: Sequencing profile of controls

Table S3: Number of reads for each sample

Table S4: Species with differential abundance across the treatment methods. Only species with a mean relative abundance of > 0.001 (0.1%) across all samples (*n* =15) were used for comparison. The p-values were calculated using the paired-sample Wilcoxon test and are FDR-adjusted: ns: not significant; .: p ≤ 0.10; *: p ≤ 0.05; **: p ≤ 0.01.

## Supplementary Figures

**
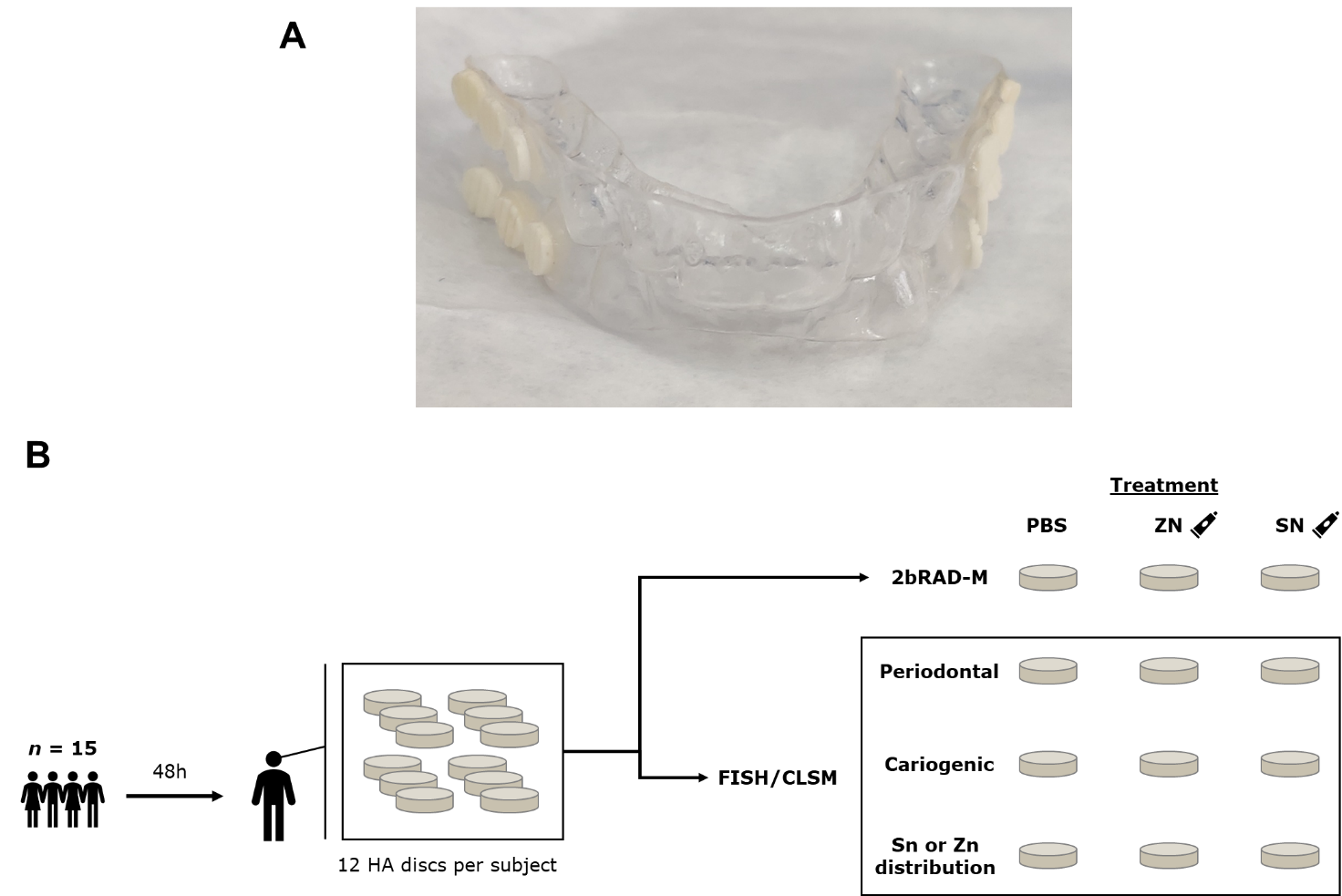
**

**Figure S1: Overview of the study design.** **A)** Photograph of the oral splint fitted with 12 hydroxyapatite (HA) disks that was used in this study to collect the in-situ plaque biofilms. **B)** Schematic of the experimental design. **
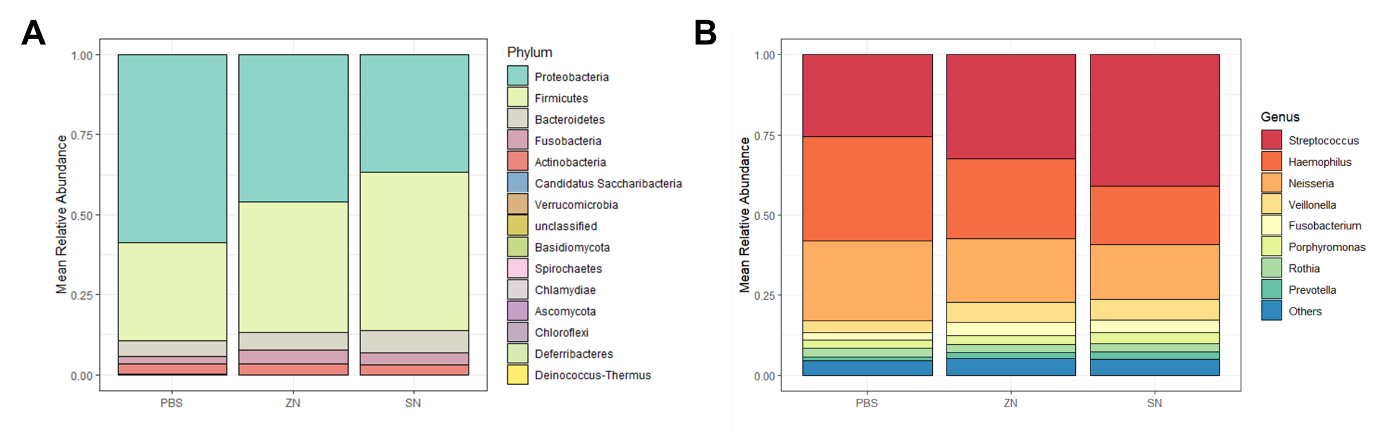
**

**Figure S2: Phylum and genus level overview of the microbial communities across the treatment groups:** Mean relative abundance of the different **A)** phyla and **B)** genera across all samples are presented. All detected phyla are shown while genera with mean relative abundance of < 0.01 (1%) are grouped under “Others”.


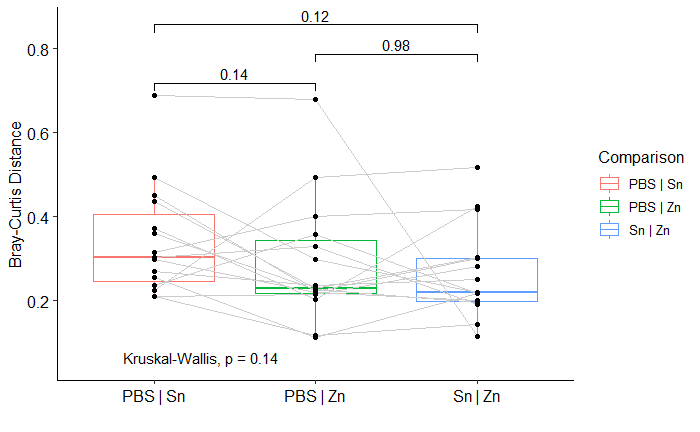


**Figure S3: Beta-diversity comparison between the different treatment groups.** The overall *p-*value was obtained using the Kruskal-Wallis test while all *p*-values above the boxplots were derived using the paired-sample Wilcoxon test.
